# PIMT Binding to C-Terminal Ala459 of CAIX Is Involved in Inside-Out Signaling Necessary for Its Catalytic Activity

**DOI:** 10.3390/ijms21228545

**Published:** 2020-11-12

**Authors:** Veronika Simko, Petra Belvoncikova, Lucia Csaderova, Martina Labudova, Katarina Grossmannova, Miriam Zatovicova, Ivana Kajanova, Ludovit Skultety, Monika Barathova, Jaromir Pastorek

**Affiliations:** 1 Department of Tumor Biology, Biomedical Research Center, Institute of Virology, Slovak Academy of Sciences, Dubravska cesta 9, 84505 Bratislava, Slovakia; virunica@savba.sk (V.S.); petra.belvoncikova@savba.sk (P.B.); lucia.csaderova@savba.sk (L.C.); virulama@savba.sk (M.L.); virukala@savba.sk (K.G.); miriam.zatovicova@savba.sk (M.Z.); viruivvi@savba.sk (I.K.); jaromir.pastorek@savba.sk (J.P.); 2 Department of Rickettsiology, Biomedical Research Center, Institute of Virology, Slovak Academy of Sciences, Dubravska cesta 9, 84505 Bratislava, Slovakia; viruludo@savba.sk; 3 Faculty of Medicine, Slovak Medical University, Limbová 12, 833 03 Bratislava, Slovakia

**Keywords:** carbonic anhydrase IX (CAIX), pH regulation, intracellular binding partner, protein-L-isoaspartyl methyltransferase (PIMT), hypoxia, cancer

## Abstract

Human carbonic anhydrase IX (CAIX), a unique member of the α carbonic anhydrase family, is a transmembrane glycoprotein with high enzymatic activity by which CAIX contributes to tumorigenesis through pH regulation. Due to its aberrant expression, CAIX is considered to be a marker of tumor hypoxia and a poor prognostic factor of several human cancers. Hypoxia-activated catalytic function of CAIX is dependent on posttranslational modification of its short intracellular domain. In this work, we have identified that *C*-terminal Ala459 residue, which is common across CAIX of various species as well as additional transmembrane isoforms, plays an important role in CAIX activation and in pH regulation. Moreover, structure prediction I-TASSER analysis revealed involvement of Ala459 in potential ligand binding. Using tandem mass spectrometry, Protein-L-isoaspartyl methyltransferase (PIMT) was identified as a novel interacting partner, further confirmed by an in vitro pulldown assay and an in situ proximity ligation assay. Indeed, suppression of PIMT led to increased alkalinization of culture media of C33a cells constitutively expressing CAIX in hypoxia. We suggest that binding of PIMT represents a novel intracellular signal required for enzymatic activity of CAIX with a potential unidentified downstream function.

## 1. Introduction

Carbonic anhydrase IX (CAIX) belongs to the α carbonic anhydrase family of zinc metalloenzymes that catalyze the reversible interconversion of carbon dioxide to form bicarbonate ions and protons [1]. In mammals, there are 16 isoforms of carbonic anhydrases, which differ in their cellular localization and activity from weak to very strong [2]. Cancer cells primarily express the plasma membrane-associated carbonic anhydrases CAIX and CAXII, as well as intracellular carbonic anhydrases such as CAI and CAII. CAIX has a unique position within this enzyme family due to its high catalytic efficiency and its strong hypoxia-related expression in a wide variety of solid tumors, including breast, colorectal, glioblastoma, lung, head and neck, and cervical cancers, typically linked with poor disease prognosis. Due to specific tumor-related expression and its role in pro-survival and pro-metastatic processes, CAIX has been extensively studied as a potential therapeutic target [3].

Transcription of the *CA9* gene is primarily regulated by the hypoxia-inducible transcription factor (HIF-1) that binds to the hypoxia response element (HRE) localized just in front of the transcription initiation site [4]. Thus, CAIX is considered as one of the best responders to low oxygen. For full transcriptional activation of *CA9*, HIF cooperates with the SP1 transcription factor which induces *CA9* by increased cell density and acidosis in a cell type-specific manner [5,6]. PI3K and ERK/MAPK signal transduction pathways, including upstream tyrosine kinases such as SRC and RET, also participate in *CA9* regulation [7,8,9]. Moreover, hypoxia regulates splicing of the *CA9* mRNA [10].

CAIX enzymatic activity is exerted by extracellularly facing the catalytic domain and is involved in cancer cell protection from intracellular acidosis, allowing tumor growth, and by creating extracellular acidosis, it promotes cancer cell migration and invasion. The CAIX-unique proteoglycan-like domain executes a non-catalytic function which is important in cell adhesion and spreading, especially in the assembly of focal adhesion contacts during cell migration [11]. Via both catalytic and non-catalytic functions, CAIX endows tumor cells with survival advantages with an increased ability to migrate, invade, and metastasize. Previously it was described that hypoxia has an impact on CAIX activity through intracellular modulation of a short cytoplasmic tail and that it depends on the phosphorylation of Thr443 residue by the protein kinase A (PKA) [12]. Complete enzyme activation also requires dephosphorylation of Ser448, but the precise mechanism has not been fully elucidated.

In this work, we continued in our investigation of intracellular tail (IC tail) and showed that the last *C*-terminal amino acid Ala459 also participates in and enhances the enzymatic activation of CAIX. Ala459 represents the last amino acid portion of the cytoplasmic tail of CAIX, which has been preserved across the CAIX of various species and also in sequences of other transmembrane carbonic anhydrases (CAXII and CAIV). Due to this structural feature we considered and explored its possible role in ligand binding. Using tandem mass spectrometry we have identified protein-l-isoaspartyl methyltransferase (PIMT) as a novel Ala459-mediated interacting partner.

PIMT is well known for its protein repair function, namely the reconversion of deamidated and/or isomerized asparaginyl and aspartyl residues into their normal, non-isomerized forms. Due to its functionality, it has been involved in the maintenance of various cellular and extracellular activities. PIMT regulates, e.g., calcium homeostasis by repairing calcium-binding protein calmodulin and thus restoring its function [13]. The role of PIMT in cell movement and tubulogenesis has also been demonstrated. In vitro knock-down of PIMT showed its important role in cell migration and microvascularization of human vascular endothelial cells (HUVEC) [14]. PIMT also prevents stress-induced damages by repairing of various antioxidant proteins, which helps the cells to escape from apoptosis. The antiapoptotic role of PIMT was studied in neuroblastoma cells where PIMT inhibited activation of caspase 3 and caspase 9 [15].

The correlation between PIMT expression levels and cancer prognosis was recently suggested [16]. Higher PIMT expression levels were related to the decreased survival of lung cancer patients following surgical intervention. Upregulation of PIMT expression in bladder cancer tissues was demonstrated [17] and correlated with the clinical stage, muscularis invasion, lymph node metastasis, and distant metastasis. The role of PIMT in cancer cell migration and invasion was also confirmed in vitro. The role of PIMT in human cancer has been partially cognized but its precise biological and clinical function in cancer remains unsolved.

In this work, we have described a new role of PIMT as a protein interacting with the intracellular tail of tumor-associated carbonic anhydrase IX and the identified terminal Ala459 in CAIX as a mediator of this interaction. Silencing of PIMT led to impairment of pH regulation function of CAIX, conveying functional importance of this interaction. Our findings point out to a new possible inside-out mechanism of the CAIX enzymatic activity regulation, involving the interaction with intracellular partners, and attract attention into the investigation of new ways of protein activity modulation.

## 2. Results and Discussion

### 2.1. Significance of C-Terminal Ala459 in CAIX-Mediated Functions

**Figure 1:**
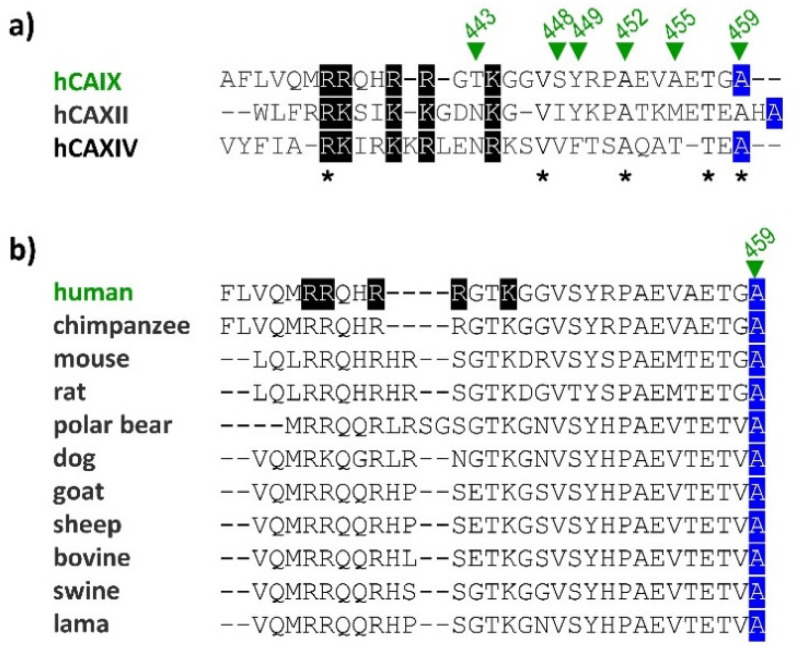
Graphical representation of amino acid sequence alignment of IC domains of human CAIX with CAXII and CAXIV (**a**) and various species of CAIX (**b**). Conserved amino acids are marked with *, conserved basic motif is marked using a black background and the last *C*-terminal Ala459 is blue-marked. Sequence alignment was analyzed using CLUSTAL Omega.

As our previous studies have revealed, an intracellular tail (IC tail) was shown as critical for the correct plasma membrane localization, which is a prerequisite for important extracellular functions of CAIX [18]. Phosphorylation of intracellular Thr443 mediated by cAMP-dependent protein kinase A (PKA) plays a key role in the catalytic activity of CAIX and controls its ability to regulate pH [12]. The PKA consensus site has been identified in the juxtamembrane basic amino acid motif within the 436-444 cytoplasmic region that was previously linked to proper functioning of CAIX. Conversion of the basic motif to a highly acidic amino acid sequence abolished the capacity of CAIX to acidify extracellular pH (pHe), inhibited binding of CAIX-selective sulfonamide inhibitor in hypoxia, and reduced shedding of the CAIX ectodomain [18]. Although basic residues (Arg and/or Lys) located within IC tail are well conserved in IC tails of two other transmembrane CA isoforms XII and XIV, Thr is present only in CAIX. The putative phosphorylation site Ser448 might act as a negative regulatory element, diminishing the ability of CAIX to acidify pHe [12]. However, a precise mechanism has not yet been elucidated. Tyr449 phosphorylation was also described and linked to downstream activation of the PI3K/Akt pathway [19], but the question of whether it affects CAIX function remains unanswered. Interestingly, the last *C*-terminal amino acid Ala459 appears to be another common structural feature among transmembrane carbonic anhydrases (Figure 1a) and is also preserved across the CAIX of various species (Figure 1b). Alanine is present in just about all non-critical protein contexts because of its very non-reactive methyl group side chain. Due to this property, it is almost never directly involved in protein function. However, it can play a role in substrate recognition or specificity, particularly in interactions with other non-reactive atoms, such as carbon [20]. Despite these properties, our preliminary data suggested the significance of Ala459 linked to pH regulation and catalytic function of CAIX.

#### Substitution of Ala459 for Gly Affects CAIX-Mediated Extracellular Acidification and Cell Migration

**Figure 2:**
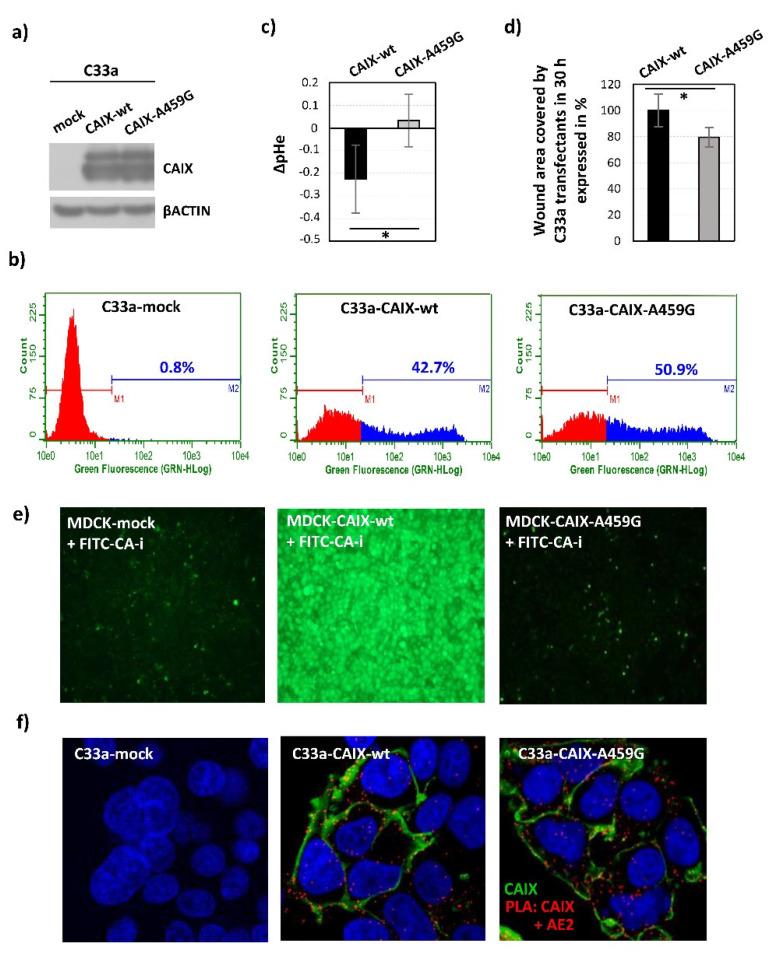
*C*-terminal amino acid Ala459 residue cooperates in the regulation of CAIX catalytic function: (**a**) Immunoblot analysis of cell lysates from C33a cells transfected with mock control, CAIX wild type (wt) or mutant (A459G) cultured 48 h in hypoxia. CAIX was detected using mouse monoclonal antibody M75 diluted 1:10 and β-actin was detected using mouse monoclonal antibody (CS3700, Cell Signaling Technology, Danvers, Massachusetts) diluted 1:5000. HRP-conjugated anti-mouse antibody (Dako Agilent, Santa Clara, California) diluted 1:5000 was used as a secondary antibody. (**b**) Fluorescence staining of CAIX in non-fixed and non-permeabilized C33a-CAIX wild type as well as A459G transfectants measured by flow cytometry. CAIX was detected using PG-domain specific mouse monoclonal antibody M75 diluted to 1 µg/mL and AlexaFluor 488-conjugated anti-mouse secondary antibody (Invitrogen, Carlsbad, California) diluted 1:1000. Results clearly demonstrate plasma membrane localization of CAIX and showed that 42.7% of C33a-CAIX-wt and 50.9% of C33a-CAIX-A459G transfectants expressed CAIX protein. C33a cells transfected with mock control plasmid were used as a negative control. The data are presented as the mean, *n* = 2. (**c**) Effect of Ala459 mutation on CAIX-mediated extracellular acidification. The graph shows the differences between pHe values (ΔpH) of culture media from CAIX wt or A459G-transfected and mock-transfected cells cultured 48 h in hypoxia. A459G mutant reduced acidification of extracellular pHe when compared to control wild type C33a-CAIX transfectants. The data are presented as the mean ± s.d., *n* = 5. Statistical significance was analyzed using the Student’s *t*-test and expressed as a *p*-value (* *p* < 0.05). (**d**) Effect of Ala459 mutation on migration capacity of C33a cells. The graph depicts the results of the wound healing assay given as a % of the area covered by cells migrating to close the wound at 30 h after the scratch, measured at various positions along the wounds. Area covered by C33a cells expressing CAIX-wt was set as 100%. C33a cells expressing CAIX with mutated Ala459 exhibited slower migration. The data are presented as the mean ± s.d., *n* = 10. Statistical significance was analyzed using the Student’s *t*-test and expressed as a *p*-value (* *p* < 0.05). (**e**) Accumulation of the fluorescent sulfonamide (FITC-CA-i) occurred in hypoxic MDCK cells expressing the CAIX-wt, whereas it was diminished in hypoxic MDCK cell transfected with the CAIX-A459G mutant. Images were taken using objective 10×. (**f**) In situ detection of the interaction between CAIX and AE2 using a proximity ligation assay (PLA). Analysis was performed in C33a cells transiently transfected with CAIX-wt and A459G mutant and cultured in hypoxia for an additional 48 h. Red PLA signal indicates the existence of interaction or close proximity localization also between CAIX with mutated Ala459 and AE2. C33a-mock transfectants served as a negative assay control due to a lack of one target protein. CAIX protein was post-labelled in green, nuclei are blue. Images were taken using objective 40× and zoom 3.

The pSG5c_CAIX-A459G construct was prepared and used for further research of *C*-terminal Ala459. C33a cervical carcinoma cells without endogenous expression of CAIX were transiently transfected with pSG5c_CAIX wild type (wt) or mutant (A459G) and cultured in hypoxic conditions for 48 h. At 72 h post-transfection we determined the protein levels of CAIX (Figure 2a) and checked the correct plasma membrane localization of mutants (Figure 2b), which allowed us to perform subsequent analyses. First, we evaluated the effect on CAIX-mediated extracellular acidification. pHe measurements of culture medium of C33a cells expressing the CAIX-A459G mutant revealed its reduced capability to acidify extracellular medium under hypoxia (Figure 2c). Acidification of extracellular space reflects the enzymatic activity of CAIX, which is also involved in the cell migration processes [21,22]. We performed a wound-healing assay and showed a significantly decreased migration rate of C33a cells ectopically expressing CAIX-A459G (Figure 2d).

Indeed, impaired catalytic function has been proven by prevented binding and accumulation of the FITC-conjugated inhibitor (FITC-CA-i), which binds only to catalytically active CAIX [12,22]. Canine MDCK epithelial cells stably transfected with pSG5c_CAIX-A459G, pSG5c_CAIX-wt and pSG5c_empty constructs were incubated in hypoxia for 48 h and binding of FITC-CA-i to living cells was visualized using fluorescence microscopy. MDCK transfectants expressing CAIX with mutated A459 showed a reduced binding capacity of FITC-CA-i (Figure 2e), which is in accordance with disabled pH regulatory function observed in C33a transient transfectants (Figure 2c).

### 2.2. Implication of C-Terminal Ala459 in the Interaction of CAIX with Potential Binding Partner(s)

We assume that the extracellular function of CAIX might be linked to allosteric changes within the IC tail and we suggest that a loss of intracellular partner(s) would impact it. In accordance with this hypothesis, substitution of Ala459 affected the capacity of CAIX to acidify the extracellular microenvironment (Figure 2c), and thereby disabled the most important extracellular function of CAIX. Abolished binding of FITC-CA-i due to impaired enzymatic activity of A459G mutant also supports this assumption (Figure 2e). This inside-out regulation could potentially disrupt a communication between the CA domain and other components of a functional metabolon, which is essential for locally concentrated production and the consequent influx of bicarbonate ions and proper pH regulation [21,23]. Forming of a transport metabolon composed of CAIX and bicarbonate transporters (AE2, NBCe1) in the lamellipodia of migrating cells enhances the ion flux through the plasma membrane and thereby promotes cell migration [11,21]. Reduced migration capacity has also been confirmed in cells expressing CAIX-A459G (Figure 2d), however the interaction between the CA domain and AE2 was detected and not disturbed (Figure 2f), suggesting that Ala459 could be implicated in proper metabolon functioning but does not affect its formation. Alternatively, structural changes at the 459 position might perturb phosphorylation of Thr443 and thereby affect CA IX activity in the acidification of extracellular pH and migration processes.

**Figure 3:**
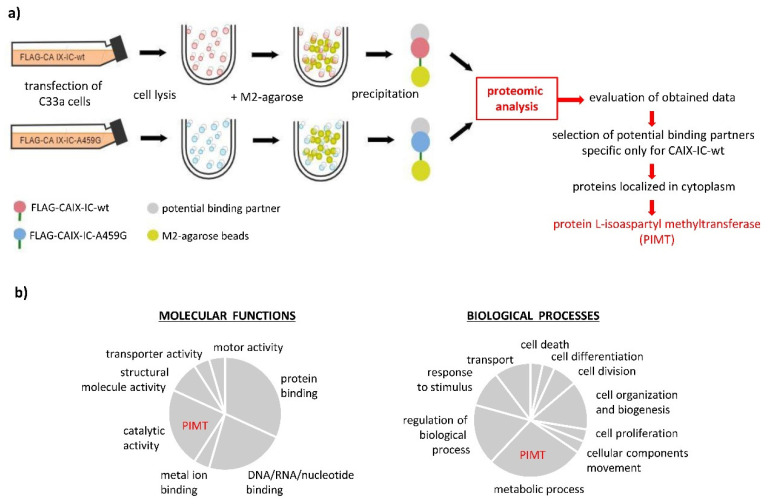
Identification of novel intracellular binding partner(s) of carbonic anhydrase IX: (**a**) Schematic outline of the experimental procedure. (**b**) Comparative analysis of proteomic data based on ProteinCenterTM analyzer (Thermo Fischer Scientific, Waltham, Massachusetts). Graphical scheme represents summary characterization and clustering of predicted binding partners selected from the comparison of wild type versus A459G sample data sets. Results proved a cluster of cytoplasmic proteins binding exclusively the wild type form of intracellular tail of CAIX (non-mutated Ala) with different molecular functions and roles in cellular processes. Among them, PIMT was characterized as a protein exhibiting catalytic activity with a function in metabolic processes.

Next, we explored the role of *C*-terminal Ala459 in a direct interaction with the so far unidentified cytoplasmic partner(s). We performed proteomic-based mapping of FLAG-tagged CAIX–IC/M2 agarose immunoprecipitates (wt versus A459G) using tandem mass spectrometry. Resulted data were then processed by ProteinLynx Global Server and interpreted by means of the ProteinCenter^™^ (Thermo Fischer Scientific, Waltham, Massachusetts). Schematic illustration of this comprehensive analysis is shown in Figure 3a. Wt versus A459G data were compared and potential cytoplasmic binding partners specific only for the non-mutated IC tail were subsequently identified. The resulting protein cluster exhibited diverse molecular functions in various biological processes summarized in pie charts depicted in Figure 3b.

### 2.3. Identification of Protein L-Isoaspartyl Methyltransferase (PIMT) as a Novel Intracellular Binding Partner of Cancer-Associated Hypoxia-Induced CAIX

Proteomic screening revealed the protein-L-isoaspartyl methyltransferase (PIMT, also known as PCMT1, encoding by *Pcmt1* gene) as a highly probable intracellular binding partner within a cluster of proteins exhibiting catalytic activity and functionality in metabolic processes (Figure 3b).

PIMT is a widely distributed enzyme functioning as a critical component of the cellular protein repair machinery [13,24,25]. Its catalytic property includes specific methylation of the isoAsp generated by the spontaneous deamidation of Asn residues accumulating in proteins during aging [26,27]. This methylation is an essential step for converting isoAsp to Asp and the transfer of the methyl group to the free carboxyl group is mediated by the binding of S-adenosyl-L-methionine (AdoMet).

The cytoplasmic portion of CAIX does not contain any Asn residues and thus cannot belong to direct substrates of PIMT. However, the replacement of Ala leads to the structural loss of a side chain methyl group, whereas Gly is the simplest amino acid with a single hydrogen atom as its side chain. That is why we suppose that this free methyl group of Ala could be possibly involved in the physical binding between PIMT and CAIX. Moreover, two other Ala residues (452, 455) are present within the CAIX-IC tail (Figure 1a). Although we have not studied yet their possible participation in pH regulation or in PIMT binding, we do not exclude this option also because of their location close to *C*-terminal Ala459.

**Figure 4:**
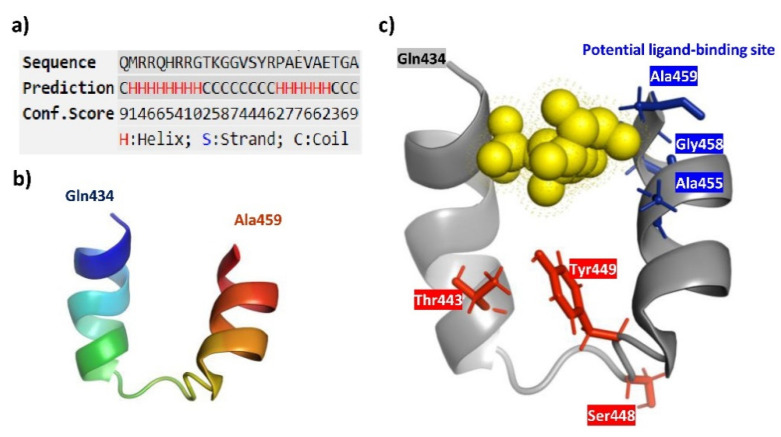
CAIX-IC domain structure prediction analyzed by a I-TASSER-based approach [28,29,30] (https://zhanglab.ccmb.med.umich.edu/I-TASSER/). (**a**) The sequence-based prediction of secondary structure of CAIX-IC domain (Q434-A459) using I-TASSER/PSSpred. Higher score means a more confident prediction of the secondary structure. (**b**) Structural conformation of predicted secondary structure. I-TASSER simulations generate a large ensemble of structural conformations called decoys. To select the final models, I-TASSER uses the SPICKER program to cluster all the decoys based on pair-wise structure similarity and reports up to five models which correspond to the five largest structure clusters. The confidence of each model is quantitatively measured by a C-score, which is calculated based on the significance of threading template alignments and the convergence parameters of the structure assembly simulations. The C-score is typically in the range of (−5, 2), where a C-score of a higher value signifies a model with a higher confidence and vice-versa. We showed and further analyzed the model with the highest C-score, which was estimated to −1.91. (**c**) Prediction of ligand-binding and residue-specific ligand binding probability of the target protein by COFACTOR and COACH based on the I-TASSER structure prediction. The five most reliable predictions were generated with the confidence score of the prediction (0–1), where a higher score indicates a more reliable prediction. One of them (with c-score estimated to 0.14) predicted Ala459, Gly458 and Ala455 as ligand binding residues. Output visualization was generated by a PyMOL Molecular Graphic System.

I-TASSER (Iterative Threading ASSEmbly Refinement) [28,29,30] analysis was initially used for the prediction of CAIX-IC domain (Q434-A459) protein structure and function. Helix-coil-helix was predicted as a secondary protein structure of CAIX intracellular tail (Figure 4a) and structural conformation was generated (Figure 4b). Based on the I-TASSER structure prediction, ligand binding sites were deduced. Analysis revealed that the predicted IC tail structure might be capable of ligand binding. Moreover, one of the five best models demonstrated the last Ala459 with Ala455 and Gly458 as probable ligand binding site residues (Figure 4c). Interestingly, predicted interaction does not affect the crucial residues Thr433, Ser448 or Tyr449. Indeed, an additional four models showed that residues localized at the 451 to 459 position can be also implicated in single ligand binding or even multiprotein cluster formation. Altogether, we conclude that *C*-terminal Ala might be a key player in PIMT binding but nearby residues could also facilitate this interaction process. We also hypothesize that *C*-terminus-mediated ligand docking could be an essential step for the correct scaffolding of the IC tail and, e.g., for subsequent proper posttranslational arrangements of critical residues, such as Thr443 and Ser488.

#### PIMT Interacts with Intracellular Tail of CAIX

**Figure 5:**
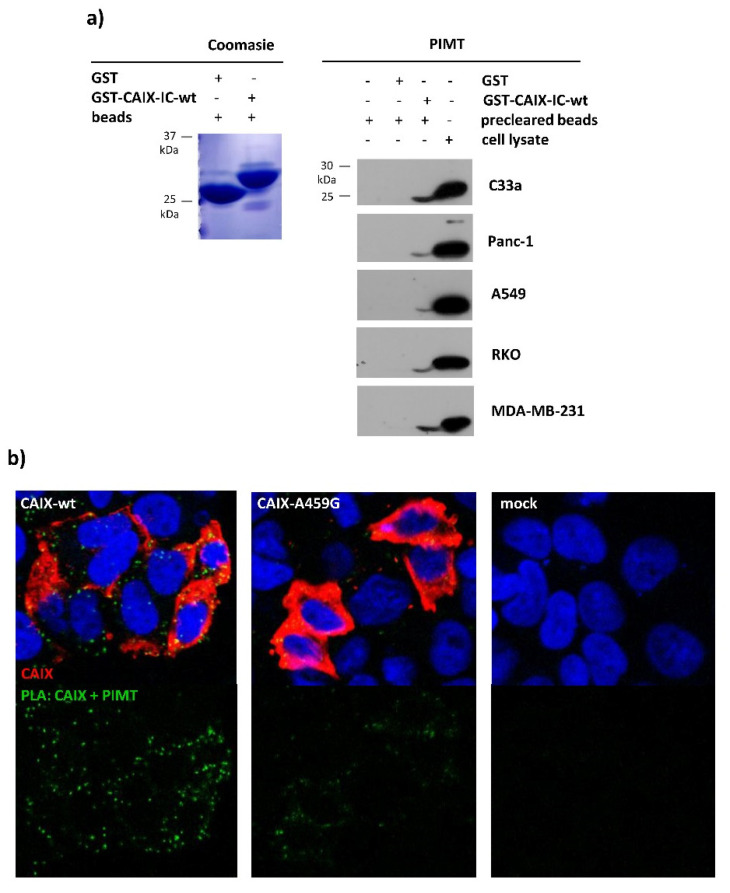
Protein L-isoaspartyl methyltransferase interacts with carbonic anhydrase IX: (**a**) Coomassie staining of purified GST and GST-CAIX-IC-wt separated by SDS-PAGE (left panel). Immunoblot analysis of GST-CAIX-IC-wt immunoprecipitates and lysates from different cancer cell lines: C33a, Panc-1, A549, RKO and MDA-MB-231 (right panel). GST immunoprecipitates and beads after lysate preclearing was used as negative control. PIMT signal was detected using monoclonal antibody (sc-100977, Santa Cruz Biotechnology, Dallas, Texas) diluted 1:1000. (**b**) In situ detection of the interaction between PIMT and CAIX protein using the proximity ligation assay. Analysis was performed in C33a cells transiently transfected with full length CAIX-wt, CAIX-A459G or mock control and cultured in hypoxia for an additional 48 h. The green PLA signal indicating the interaction of CAIX with PIMT was clearly visible in the cells expressing wild type CAIX, while in CAIX-A459G the extent and intensity of the signal was considerably lower. C33a-mock transfectants served as a negative assay control due to a lack of one target protein. CAIX protein was post-labelled in red, nuclei are blue. Images were taken using objective 40× and zoom 3.

In this study, we identified PIMT as a binding partner of cancer-associated hypoxia-induced CAIX and we confirmed their physical interaction by pull-down assay (Figure 5a) and proximity ligation assay (PLA, Figure 5b). GST-tagged CAIX-IC-wt recombinant protein was produced, isolated, immobilized on glutathione sepharose beads (Figure 5a, left panel) and incubated with hypoxic cell lysate. Using this approach, we showed the binding capacity of PIMT protein expressed by cells of various cancer types and recombinant intracellular domain of CAIX (Figure 5a, right panel). To visualize the interaction between PIMT and CAIX in hypoxic cell culture, we used PLA that allowed us to detect endogenous protein complexes in situ [31]. Using antibodies specific for the TM-IC region of CAIX and for cytoplasmic PIMT protein, we detected a strong PLA signal in hypoxic C33a cells ectopically expressing full length membrane-localized wild type CAIX (Figure 5b), but not in the CAIX-negative mock control cells. Interestingly, the PLA signal between CAIX with A459G mutation and PIMT protein was distributed in a lesser extent and exhibited a low intensity. These results indicate that certain weak binding affinity of PIMT protein to CAIX-A459G mutants might be possible and point to a possibility that the adjacent part of the CAIX IC tail, particularly Ala452 and/or Ala455, could also be engaged in PIMT binding as we have discussed previously.

### 2.4. Cancer-Related Expression and Function of Pcmt1/PIMT

PIMT is widely distributed across mammalian tissues, with particularly high levels in the CNS [32,33,34], and it plays a critical role in the maintenance of protein integrity. Complete loss of PIMT has harmful consequences [26]. Deficiency of this enzyme resulted in the accumulation of altered proteins, retardation of growth and fatal epileptic seizure in mice [35,36]. In humans, single nucleotide polymorphisms in gene encoding PIMT (*Pcmt1*) have been associated with a risk of spina bifida and premature ovarian failure [37]. Because of the relevance of PIMT, most studies have investigated its physiological functions linked to aged protein repair or its role in the pathogenesis of Alzheimer’s disease [38].

**Figure 6:**
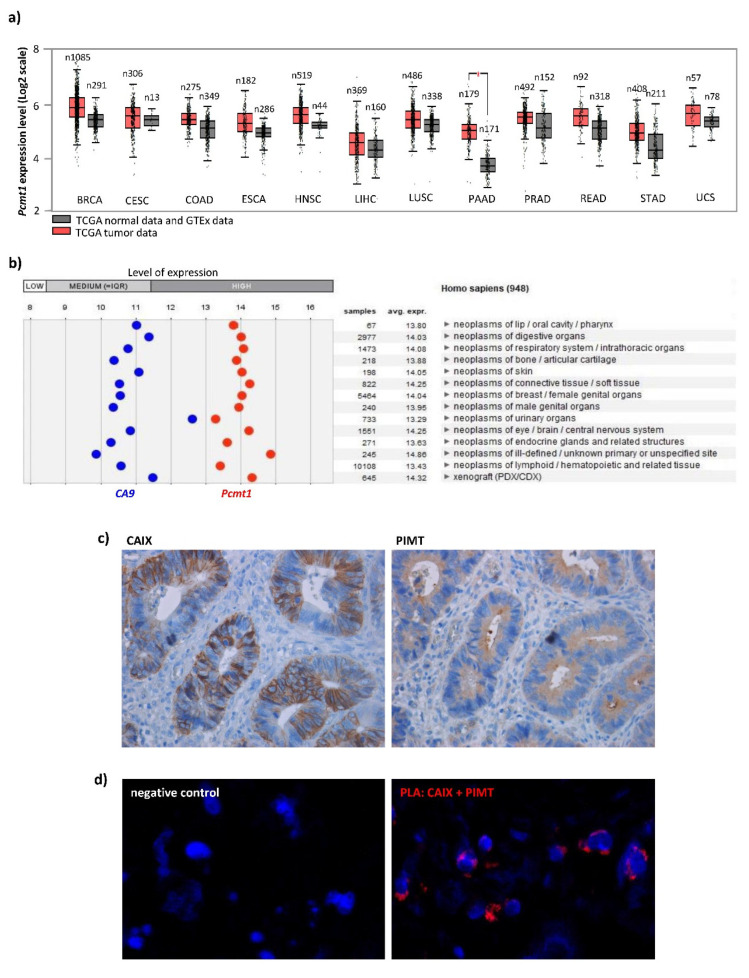
Cancer-associated expression of *Pcmt1*/PIMT: (**a**) Output from GEPIA analysis of *Pcmt1* expression in healthy versus tumor tissues (http://gepia.cancer-pku.cn/about.html) [40]. GEPIA is a web server for analyzing the RNA sequencing expression data of 9736 tumors and 8587 normal samples from the TCGA and the GTEx projects, using a standard processing pipeline. Increased tumor-associated *Pcmt1* levels were shown in a wide spectrum of cancer-types: BRCA-breast invasive carcinoma, CESC-cervical squamous cell carcinoma and endocervical adenocarcinoma, COAD-colon adenocarcinoma, ESCA-esophageal carcinoma, HNSC-head and neck squamous adenocarcinoma, LIHC-liver hepatocellular carcinoma, LUSC-lung squamous cell carcinoma, PAAD-pancreatic adenocarcinoma, PRAD-prostate adenocarcinoma, READ-rectum adenocarcinoma, STAD-stomach adenocarcinoma, UCS-uterine carcinosarcoma. Data were expressed in Log2 scale, *n* = number of samples. (**b**) Output from GENEVESTIGATOR analysis of *Pcmt1* and *CA9* gene expression levels in various cancer types (https://genevestigator.com/). Data were selected from HS_AFFY_U133PLUS_2-0, analyzed using Cancer/ScatterplotTree settings and expressed as Log2. (**c**) Representative image of immunohistochemical staining of CAIX and PIMT in CRC patient tissue sample. Images were taken using objective 40×. (**d**) Representative image showing the interaction between PIMT and CAIX protein in CRC patient tissue samples detected by proximity ligation assay. Red PLA signals indicating the interaction of CAIX with PIMT, nuclei are blue. Negative control was prepared by omission of the PIMT primary antibody. Images were taken using objective 40× and zoom 3.

Recent studies linked PIMT with cancer phenotypes, motility and migration of cancer cells in vitro [17,39], suggesting its role in tumor metastasis dissemination. Indeed, increased expression levels of PIMT was detected in various cancer types and was associated with advanced clinical stages, correlated with lymph node, distant metastases and poorer survival of patients [16,17]. We performed Gene Expression Profiling Interactive Analysis (GEPIA) [40], which allowed us to compare the expression of *Pcmt1* in various cancer types versus corresponding normal healthy tissues and showed higher levels of *Pcmt1* in tumor-derived samples (Figure 6a). GENEVESTIGATOR analysis of microarray data also proved the expression of *Pcmt1* in a broad spectrum of *CA9-*positive cancers (Figure 6b). We stained both proteins in CRC tissue samples and showed co-localization of PIMT with CAIX (Figure 6c). Indeed, proximity ligation assay performed in the CRC tissue samples showed interaction of PIMT and CAIX (Figure 6d), giving supporting evidence for the existence of their mutual interaction in vivo.

Higher levels of PIMT are in agreement with its physiological role in damaged protein repair that might contribute to cancer cell survival. In addition, it was published that PIMT suppressed the proapoptotic and checkpoint activation functions of p53 in various cancer cells in vitro. PIMT decreased activity of p53 through direct methylation and also interacted with p53, physically causing destabilization of p53 by enhancing the p53/HDM2 interaction [41]. Further study showed that calcium binding protein S100A4 promotes proteasome-mediated degradation of p53 by a mechanism which requires its complexing with PIMT [42]. S100A4 (known as metastasin) also plays an important role in human cancer development, progression, and metastasis [43,44,45] and an increased expression level of S100A4 in various cancer types was associated with hypoxia-induced invasion due to direct transcriptional activation by HIF-1α [46,47].

#### Hypoxia Contributes to the Expression of PIMT

Hypoxia resulting from an inadequate oxygen supply is a characteristic feature of a wide range of solid tumors where it associates with aggressive malignancy, poor prognosis, and resistance to therapy. Hypoxia-inducible factor-1 (HIF-1) has been identified as a master regulator of cell adaptation to hypoxic conditions through the transcriptional activation of many genes, including *CA9*, and thus controlling a broad spectrum of cancer-related processes, such as angiogenesis, cell survival, and cell invasion [48]. High cancer-associated levels of PIMT/*Pcmt1* as well as the direct interaction of PIMT with hypoxia-induced CAIX (or metastasin), prompted us to clarify whether hypoxia could participate in the regulation of the *Pcmt1* gene.

**Figure 7:**
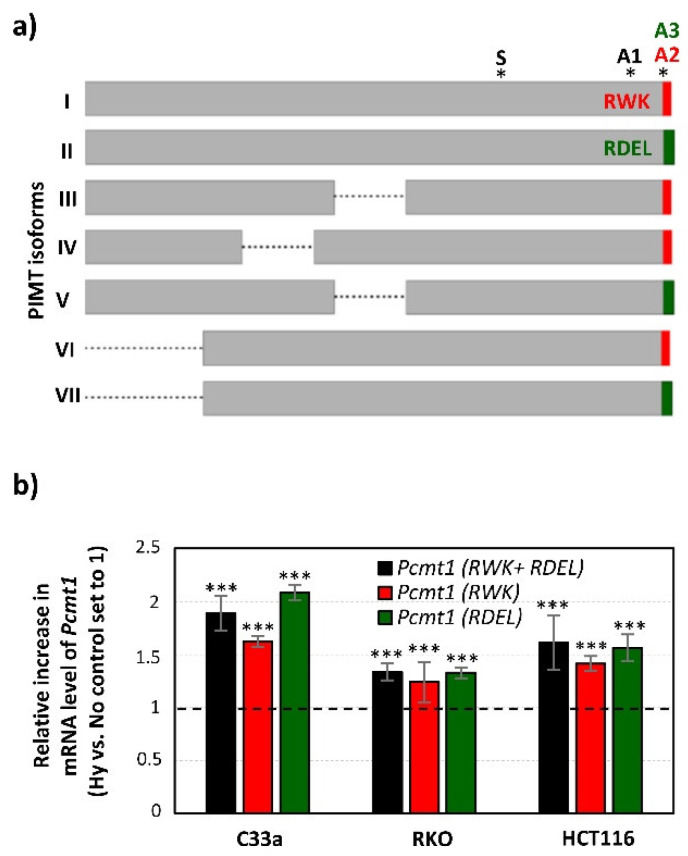
Hypoxia contributes to the regulation of *Pcmt-1* gene expression: (**a**) Graphical alignment of PIMT protein isoforms with denoted location of oligonucleotides used in qPCR (Figure 7b). Schematic localization of *Pcmt1*-specific oligonucleotides (S, A1, A2, A3) are depicted with *. The description of each PIMT isoform is as follows: Isoform I encoded by transcript variant 1 represents the longest isoform. Isoform II (as a product of transcript variant 2) has a longer and distinct *C*-terminus due to a frameshift caused by alternative splice site in the 3′ coding region. Isoform III and IV are shorter when compared to isoform I due to a lack of coding exon in transcript variant 3 and 4. Transcript variant 5 also lacks an in-frame coding exon and uses an alternate splice site in the 3′ coding region, which results in a frameshift and thus the encoded isoform V is shorter and has a distinct *C*-terminus when compared to isoform I. Transcript variant 6 differs in 3′ UTR but encodes the same isoform as variant 1 (isoform I). Isoform VI has a shorter *N*-terminus, because its encoding transcript variant 7 has the same structure as 1, but a different 5′ UTR and uses an alternative start codon. Similarly, isoform VII has a shorter *N*-terminus, but with a distinct *C*-terminus, because transcript variant 8 uses an alternate splice site in the 3′CDS, resulting in a frameshift. (**b**) Quantitative PCR analysis of mRNA levels of either all *Pcmt-1* transcript variants or variants encoding RWK/cytoplasmic type or RDEL/ER type isoforms. The graph shows changes in mRNA levels in hypoxic (Hy) versus normoxic (No) samples normalized to actin. The data are represented as the mean ± s.d., *n* = 3. Statistical significance was analyzed using the Student’s *t*-test and expressed as a *p*-value (*** *p* < 0.005).

Alternative splicing of *Pcmt1* genes produces multiple transcript variants (1-8, provided by NCBI Reference Sequence Database: RefSeq) encoding seven isoforms (I-VII). Schematic comparison is shown in a Figure 7a and their structural characterizations are thoroughly described in the legend. Briefly, two types of PIMT isoforms with various lengths arise due to alternative splicing, which differ in *C*-terminal amino acids. The more basic isoforms carry a RWK sequence and are cytoplasmic, while the more acidic isoforms bear a RDEL sequence which was previously identified as a potential trafficking signal responsible for endoplasmic reticulum (ER) retention and were thus denoted as ER-resident [49].

In this study, we explored the expression of PIMT during 1% O_2_ hypoxia in cervical C33a and colorectal RKO and HCT116 cancer cells at transcriptional levels. We determined the mRNA level of *Pcmt1* using quantitative PCR (qPCR). Our results showed the increased mRNA level of *Pcmt1* (both cytoplasmic and ER-types) in all cell lines when compared to a normoxic control (Figure 7b). Moreover, we showed that hypoxia induced levels of both mRNA types, detected as an mRNA pool encoding RWK-cytoplasmic type isoforms (transcript variant 1, 3, 4, 6 and 7) or a pool encoding RDEL-ER type isoforms (transcript variant 2, 5 and 8).

Although several studies investigated the role of PIMT, little is known about the mechanisms that regulate PIMT in cells. DeVry and colleagues [50] revealed that the human *Pcmt1* gene contains potential cis-acting elements, such as antioxidant (ARE), xenobiotic (XRE), and estrogen (ERE) response elements, suggesting that PIMT may be regulated by stresses, environmental factors and hormones. However, no experimental data confirm these assumptions. Several studies showed that a non-hydrolysable analog of guanosine-5′-triphosphate stimulates isoAsp methylation in various cell types [51,52]. Such a compound acts as a potent adenylyl cyclase stimulator and indicates that G-protein/adenylyl cyclase and downstream cAMP/PKA signaling could participate in the regulation of *Pcmt1*. We performed in silico analysis of the human *Pcmt1* gene upstream region using a MatInspector (https://www.genomatix.de/) in order to investigate the putative transcription factor (TF) binding sites. Analysis showed multiple cAMP-responsive binding protein (CREB) sites and thus supports the role of the cAMP/PKA/CREB axis in the regulation of the *Pcmt1* gene. Moreover, the cAMP/PKA pathway is activated by hypoxia at several levels such as upregulation of catalytic and regulatory subunits of PKA [12,53]. We have previously described the enhanced accumulation of intracellular cAMP during hypoxia due to the transcriptional activation of adenylyl cyclase 6 and 7 [54]. CREB is also considered as a hypoxia-responsive transcription factor. In accordance with hypoxia-induced levels of *Pcmt1* (Figure 7b), putative binding sites for HIF-1α/ARNT heterodimers were also detected by MatInspector as well as binding sites for other hypoxia-responsive TFs (such as AP1, SP1, EGR, ETS, SMAD, NFκB). Although we did not elucidate the precise mechanism, we can conclude that hypoxia is involved in the regulation of PIMT and may contribute to its cancer-related functions.

### 2.5. Significance of the Interaction between CAIX and PIMT

**Figure 8:**
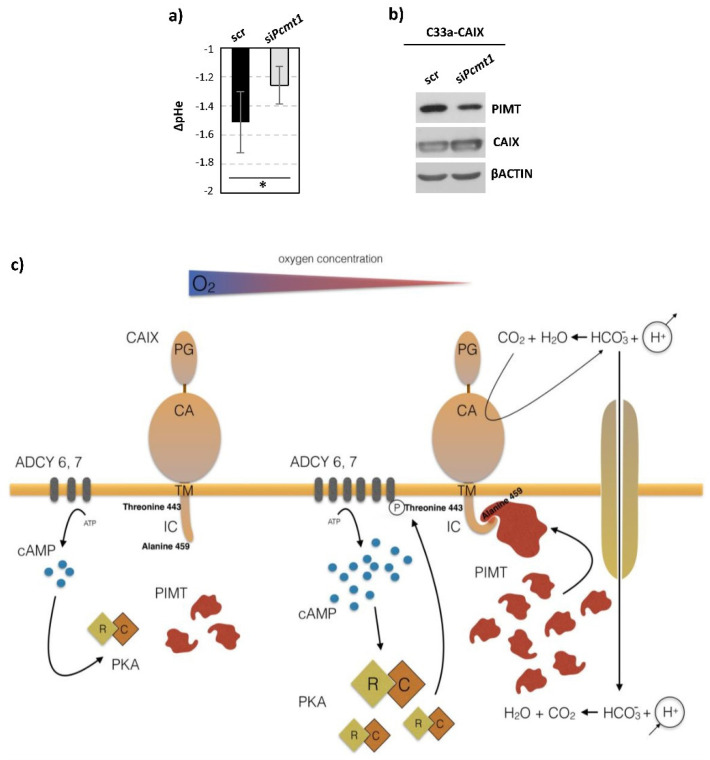
Functional role of PIMT binding to CAIX: (**a**) Effect of *Pcmt1* transient silencing on CAIX-mediated extracellular acidification. The graph shows the differences between pH values (ΔpH) measured in culture medium at the beginning and after 48 h in hypoxia normalized to protein concentration. Results clearly showed that transient *Pcmt1* silencing in C33a-CAIX cells (stable CAIX transfectants) resulted in the alkalinization of extracellular pHe when compared to scramble control (scr). Measurements were performed after 72 h post-transfection of siRNAs. The data are presented as the mean ± s.d., *n* = 6. Statistical significance was analyzed using the Student’s *t*-test and expressed as a *p*-value (* *p* < 0.05). (**b**) Efficacy of transient silencing was checked in parallel at the protein level using immunoblot analysis depicted on a representative image. (**c**) Activation of CAIX enzymatic function under hypoxic conditions. Posttranslational modification of the intracellular tail is necessary for full activation of CAIX enzymatic function. Phosphorylation of Thr443 was identified previously. In this work we found out a novel condition necessary for CAIX activation—interaction between the IC tail of CAIX and PIMT facilitated by terminal Ala459. Hypoxia can affect the activity of CAIX in several ways: it upregulates cAMP levels through ADCY6 and ADCY7, leading to higher levels of cAMP, PKA activation and Thr443 phosphorylation, and also enhances the expression of PIMT, allowing its interaction with the IC tail of CAIX.

The most important aspect of this study is the evidence of the functional role of intracellular CAIX-PIMT-composed interactome in the extracellular pH regulation. We studied the effect of PIMT inhibition provided by transient silencing of the *Pcmt1* gene, on CAIX-mediated extracellular acidification. Our results clearly show that decreased levels of PIMT (Figure 8b) in C33a cells constitutively expressing human CAIX resulted in reduced acidification of the extracellular microenvironment after 48 h in hypoxia when compared to scramble control (Figure 8a). Thus, we conclude that presence of PIMT represents the fundamental aspect for proper CAIX enzymatic activity.

We hypothesize that this interaction might be crucial for the structural scaffold of the IC domain, allowing posttranslational modifications of critical residue Thr443, or even provide a protective structural packaging. It is not clear whether the full enzymatic activity of PIMT is needed for this interaction following pH regulation, and also whether the formation of this functional interactome might act as an intracellular gating signal for CAIX extracellular enzymatic activity (Figure 8c). Thus, further investigations are certainly necessary.

This study also opens a new insight on CAIX functionality and its possible direct involvement in intracellular events. These processes might also be governed by a more complex interactome, in which PIMT can act as a structural and functional mediator by means of which CAIX exhibits its pro-tumorigenic functions inside cancer cells.

## 3. Materials and Methods

### 3.1. Cell Culture

Human C33a cervical carcinoma cells (ATCC HTB-31), HCT116 (ATCC CCL-247) and RKO (ATCC CRL-2577) colorectal carcinoma cells, MDA-MB-231 breast carcinoma cells (ATCC HTB-26), A549 lung carcinoma cells (ATCC CCL-185), Panc-1 (ATCC CLR-1469) pancreatic carcinoma and MDCK canine epithelial cells (ATCC CCL-34) were cultured under standard conditions in Dulbecco’s modified Eagle’s medium (BioSera, Nuaille, France) supplemented with 10% fetal calf serum (BioWhittaker, Basel, Switzerland) and gentamicine (Sandoz, Holzkirchen, Germany) in humidified air containing 21% O_2_, 5% CO_2_ at 37 °C and in hypoxic conditions at anaerobic workstation (1% O_2_, 2% H_2_, 5% CO_2_, 91% N_2_, Ruskinn Technology, Bridgend, United Kingdom).

### 3.2. Plasmids

In vitro mutagenesis of Ala459 to Gly was carried out by inverse PCR using the pSG5c_CAIX expression plasmid as a template [1]. A portion of *CA9* cDNA representing the IC tail (nt 1,345–1,419) or its mutated variants was cloned into pFLAG-CMV6a or pGEX-4T1 plasmid (GE Healthcare, Chicago, IL, USA) from pSG5c_CAIX [12].

### 3.3. Cell Transfection

#### 3.3.1. Transient Transfection

The C33a cells were plated into 60 mm Petri dishes to reach approximately 70% monolayer density on the following day. Transfection of 4 µg plasmid DNA was performed using Turbofect reagent (Thermo Fischer Scientific, Waltham, MA, USA) according to the manufacturer’s recommendations. After 24 h, the transfected cells were trypsinized, plated according to the type of planned experiment, allowed to attach and cultured in normoxia and hypoxia for 48 h. For silencing of the human *Pcmt1* gene, the C33a-CAIX cells were plated into 30 mm Petri dishes and transfection of 1.5 µg of MISSION esiRNA (human *Pcmt1* EHU041991 or *Rluc* EHURLUC as negative scramble control (scr) (both from Sigma-Aldrich, Saint-Louis, MO, USA), was performed using the MISSION esiRNA transfection reagent (S1452, Sigma-Aldrich, Saint-Louis, MO, USA). On the following day, cells were moved into a hypoxic station and cultured for an additional 48 h.

#### 3.3.2. Stable Transfection

MDCK cell line constitutively expressing the wild type (wt) CAIX protein or its A459G mutant were obtained by co-transfection of pSG5c_CAIX-wt, pSG5c_CAIX-A459G plasmids with pSV2neo plasmid in a 10:1 ratio using a Turbofect reagent. Transfected cells were selected in medium containing G418 (500 µg/mL). The cells cotransfected with empty pSG5c plasmid and pSV2 neo were used as mock controls. C33a cell line constitutively expressing CAIX protein (C33a-CAIX) was prepared as described in [55].

### 3.4. Western Blotting

Cells were rinsed twice with PBS, resuspended in ice-cold lysis buffer (0.1% deoxycholic acid, 1% Triton X-100 in PBS) containing a protease (Roche, Basel, Switzerland) and phosphatase inhibitors cocktail (Sigma Aldrich, Saint-Louis, MO, USA) and cleared by centrifugation. Protein concentrations were quantified using the BCA protein assay reagents (Pierce, Waltham, MA, USA). The extracts (40 µg/lane) were resolved in 10% SDS-PAGE and transferred to a PVDF membrane (Macherey-Nagel, Düren, Germany). Protein bands were visualized using an enhanced chemiluminescence kit (GE Healthcare Bio-Sciences, Chicago, Illinois).

### 3.5. Flow Cytometry

After 48 h of hypoxic incubation, transiently transfected cells were scraped into culture medium, centrifuged at low speed, washed twice with Versene solution and incubated with the CAIX-specific mouse monoclonal M75 antibody (1 µg/mL, BMC SAS, Bratislava, Slovakia) for 30 min at 4 °C. Following the centrifugation and washing with Versene, cells were incubated with the secondary anti-mouse Alexa Fluor 488-conjugated antibody (Invitrogen, Carlsbad, California) diluted 1:1000 in 1% BSA for 30 min at 4 °C. Pelleted cells were again washed twice with Versene and finally analyzed using a Guava easyCyte plus flow cytometer (Millipore, Darmstadt, Germany). Data were analyzed with Cytosoft 5.2 software using Guava Express Pro (Millipore, Darmstadt, Germany). Debris, cell doublets and clumps were excluded from analyses by scatter gating, and a total of 10,000 single cells were analyzed for each sample.

### 3.6. pH Measurement

pH of cell culture media was measured by a special microelectrode (Mettler Toledo, InLab^®^ Micro, Bratislava, Slovakia) designed for measurement in small volumes. The measurement by the electrode was done directly in the hypoxic workstation under the oxygen concentration of 1%.

### 3.7. Wound Healing Assay

C33a cells were seeded into 12-well plates and transfected with pSG5c_CAIX-wt and pSG5c_CAIX-A459G plasmids. 24 h post transfection, cells were moved into a hypoxic workstation and cultured for 24 h. As a next step, the cells were starved in DMEM with 0.5% FCS for 6 h in hypoxia. A wound was made with a sterile micropipette tip. Floating cells were removed by washing with PBS. Fresh DMEM with 0.5% FCS and a hepatocyte growth factor (HGF, 5 ng/mL, Sigma) was then added. Time-lapse acquisition was performed at a Zeiss Cell Observer System at a magnification of 100×, in the incubation chamber at 37 °C in 2% O_2_ and 5% CO_2_ atmosphere. Imaging was managed by Axiovision 4.8 software, using the Multidimensional Acquisition settings. We evaluated the movement of the migrating fronts of cells covering the wound at 5 positions (for each sample). Positions were selected at a sufficient distance from each other and their fields of view never overlapped. Wound healing was quantified using ImageJ software as the wound area covered by cells in 30 h and results were compared by *t*-test.

### 3.8. Binding of Fluorescent FITC-Conjugated Inhibitor

The fluorescent sulfonamide FITC-CA-i was obtained from homosulfanilamide and fluorescein isothiocyanate was used as described previously [18,22]. The cells were incubated for 48 h in hypoxia, and the binding of the FITC-CA-i to living cells was viewed by a Nikon E400 epifluorescence microscope.

### 3.9. Protein Identification by Tandem Mass Spectrometry

For identification of possible binding partner(s) of IC-tail of CAIX, C33a cell were transfected with pFLAG_CAIX-IC-wt and pFLAG_CAIX-IC-A459G and cultured in hypoxia for 24 h. Protein extracts were prepared using lysis buffer (0.1% deoxycholic acid, 1% Triton X-100 in PBS) and FLAG-tagged proteins were bound to FLAG-specific M2-agarose (Sigma-Aldrich, Saint-Louis, MO, USA) and washed. The proteins were eluted by 0.1 M glycine HCl with pH 3.5 after 5 min incubation at room temperature and then reduced by 10 mM dithiothreitol (DTT, Sigma-Aldrich, Saint-Louis, MO, USA) in 25 mM NH_4_HCO_3_ for 45 min at 56 °C and alkylated in the dark at room temperature for 45 min in the same buffer containing 55 mM iodoacetamide (IAA, Sigma-Aldrich, Saint-Louis, MO, USA). After neutralization of IAA, 10 µL of trypsin solution (containing 40 ng of lyophilized sequencing grade modified trypsin dissolved in 50 mM NH_4_HCO_3_; Promega, Madison, WI, USA) was added, and the proteins were digested overnight at 37 °C under gentle shaking. Enzymatic cleavage was terminated by 1 µL of 10% formic acid (Sigma-Aldrich, Saint-Louis, MO, USA). Peptides were concentrated to a volume of 20 µL and stored at −80 °C until MS analysis. Tryptic peptides were injected at a flow rate of 350 nL/minute into the nanoAcquity UPLC column (BEH 130 C18, 75 µm × 150 mm, 1.7 µm particle size). The UPLC column was directly connected to the quadrupole time-of-flight mass spectrometer (Q-TOF Premier, Waters, Milford, Massachusetts) through the nanospray emitter (3.4 kV at 70 °C). During the UPLC, an acetonitrile gradient (10–45% containing 0.1% formic acid) was used. During data acquisition, scan rates were set to 1 s with a 0.05-s interscan delay. External mass calibrant Glu-1-fibrinopeptide B (500 fmol/µL) was infused through the reference line at a flow rate of 500 nL/min and sampled every 30 s. The collision energy of 20 eV was used to collect calibrant data. Alternating low (3 eV) and elevated collision energy modes (20–38 eV) were used to collect MSE data. These data were then processed by ProteinLynx Global Server v. 3.0.3 (PLGS, Waters, Milford, MA, USA). Precursors and fragment ions were paired using correlations of chromatographic elution profiles in low/high energy traces. For protein identification, the Ion Accounting search algorithm was applied. The reference sequence file downloaded from https://www.uniprot.org/proteomes/UP000005640 contained 73101 protein sequences. The workflow parameters for the protein identification searches were as follows: one possible missed cleavage utilizing trypsin as the protease, fixed modification of Cys (carbamidomethylation), possible modifications of Met (oxidation) and Asn/Gln (deamidation). The precursor and peptide fragment mass tolerances were automatically determined by the software. Protein identification was limited to less than a 4% false discovery rate against the randomized database, applied at the individual peptide level. Identifications were accepted if at least two distinct reliable peptides matched the protein sequence.

### 3.10. Pull Down Assay

The proteins GST-CAIX-IC or GST (as control sample) were expressed in Escherichia coli BL21. After induction for 4 h with 0.2 mM IPTG at 30 °C, the cells were harvested and lysed. After sonication and centrifugation, the supernatants were mixed with washed Glutathione Sepharose 4B beads (GE Healthcare, Chicago, IL, USA) and rotated overnight at 4 °C. Cancer cell extracts were prepared from cells cultured 48 h in hypoxia plated on 100 mm Petri dishes, lysed using standard protocol (described in Western blotting) and precleared with Glutathione Sepharose 4B beads. 10 µg of GST and GST-CAIX-IC proteins bound on beads were than incubated with precleared cell extracts overnight at 4 °C, washed five time with PBS, boiled for 5 min in 40 µL sample buffer containing mercaptoethanol and separated by SDS-PAGE. After Western blotting, presence of precipitated PIMT in samples containing GST-CAIX-IC was analyzed using specific monoclonal antibody (sc-100977, Santa Cruz Biotechnology, Dallas, TX, USA) diluted 1:1000. GST/cell extracts precipitates and beads after cell extracts preclearing served as negative controls. PIMT protein was simultaneously verified in cell extracts (40 µg per lane).

### 3.11. Immunohistochemistry

Colorectal carcinoma (CRC) tissues were fixed with formalin after surgery and were processed for paraffin embedding. Paraffin-embedded tissues were sectioned and mounted onto glass slides. The slides were deparaffinized and rehydrated. CAIX and PIMT were detected using the EnVision^™^ FLEX System (DAKO Agilent, Santa Clara, CA, USA) according to the instructions. Sections were incubated with anti-CAIX antibody M75 (diluted 1:100) or anti-PIMT antibody (NB100-417, Novus Biological, Centennial, Colorado; diluted 1:300) one hour at room temperature. Negative controls were prepared by omission of the primary antibody. The stained sections were examined and photographed with Leica DFC 480 microscope. The study protocol was approved by the Ethics Committee of Biomedical Research Center, Slovak Academy of Sciences, Ethics approval EK/BmV-02/2016 and was in accordance with the principles of the Declaration of Helsinki. All subjects gave their written informed consent before participation.

### 3.12. Proximity Ligation Assay

Proximity ligation assay was performed in a humid chamber at 37 °C according to the manufacturer’s instructions (Duolink In Situ detection reagent-Green, Sigma Aldrich). Cells were seeded on glass coverslips and allowed to attach and transferred to hypoxia. They were then fixed with 4% paraformaldehyde and permeabilized with 0.2% Tween-20 in PBS. Paraffin-embedded CRC tissues (Ethics approval EK/BmV-02/2016) were deparaffinized, rehydrated and antigen retrieval was performed using citrate unmasking solution. Samples were blocked with 3% BSA/PBS for 30 min, incubated with a mixture of antibodies against CAIX (NB100-417, Novus Biological, Centennial, Colorado; diluted to 1 µg/mL) and PIMT (sc-100977, Santa Cruz Biotechnology, Dallas, TX, USA; diluted to 2 µg/mL) or CAIX (M75, diluted to 1 µg/mL) and AE2 (GenScript, Piscataway, NJ, USA; diluted to 1 µg/mL) for 1 h, incubated with plus and minus PLA probes for 1 h, then incubated with a ligation mixture containing connector oligonucleotides for 30 min, and finally with an amplification mixture containing a fluorescently labelled DNA probe for 100 min. The samples were analyzed by laser scanning confocal microscopy (LSM 510 Meta Microscope; Zeiss, Oberkochen, Germany). All PLA stainings were performed in two independent experiments and representative images of each analysis are shown in figures.

### 3.13. Quantitative PCR

Total RNA was isolated using Trizol solution (Sigma-Aldrich, Saint-Louis, MO, USA) and reverse transcription of 2 μg RNA for each sample was performed with the High-Capacity cDNA Reverse Transcription kit (Applied Biosystems, Foster City, CA, USA) according to the manufacturer’s recommendations. qPCR was carried out using Maxima Syber Green PCR Master mix (Thermo Fisher Scientific, Waltham, MA, USA) and ran for 10 min at 95 °C for initial denaturation followed by 40 cycles of 95 °C for 15 s and 60 °C for 1 min. Sample Ct values were normalized to actin. Relative expression was calculated using the ΔΔCt method. All amplifications were performed in triplicate. Results were calculated from three independent experiments. Oligonucleotide sequences (5′-3′) used for qPCR are as follows: *Actin*_S: ccaaccgcgagaagatgacc, *Actin*_A: gatcttcatgaggtagtcagt, *Pcmt1_S:* gcttgttgtgggggatggaa, *Pcmt1_A1*: cccccatcagaggcttcattt, *Pcmt1_A2*: aatcacttccacctggaccac *Pcmt1_A3*: cttttacaattcatccctggaccac.

## 4. Conclusions

In this work, for the first time we identified *C*-terminal Ala459 residue as an element essential for proper enzymatic activity of CAIX. We showed that a structural change at 459 position perturbs the ability of CAIX to regulate pH, decreases its enzymatic activity and thus affects the cell migration. Moreover, structural prediction of intracellular tail conformation indicated a potential role of Ala459 in ligand binding, suggesting a possibility of the existence of so the far unidentified cytoplasmic partner. Using a proteomic-based approach, we found and subsequently proved the intracellular interaction of CAIX with cytoplasmic protein PIMT. Indeed, Ala459 seems to be the key structural component of the IC domain facilitating this bond, but other *C*-terminal residues may also be involved in PIMT binding. In the last decade, PIMT has been studied in the context of cancer development and progression and it appears to be an effective participant in metastatic processes. Furthermore, PIMT is expressed at a high level in a broad range of tumors. Limited evidence is available about the regulation of *Pcmt1* gene expression, nonetheless we identified that tumor hypoxia might contribute to its enhanced cancer-linked expression. Elucidation of the mechanisms of how hypoxia regulates alternative splicing of PIMT mRNA could provide important data on the specific roles of PIMT in cancer and should be included in future studies. In this work, we provide the evidence that PIMT functionally contributes to tumor microenvironment through the CAIX-mediated pHe regulation. Finally, we conclude that the physical interaction between *C*-terminal Ala459 localized in the IC tail of CAIX and PIMT, driven by tumor hypoxia, may act as a novel intracellular prerequisite for full CAIX enzyme activation and determination of its entire functional significance and clinical relevance deserves concentrated investigation in the future.

## Abbreviations

AdoMet	S-adenosyl-L-methionine
AE2	anion exchange protein 2
AP1	activator protein 1
ARE	antioxidant response elements
ARNT	aryl hydrocarbon receptor nuclear translocator
CAIV	carbonic anhydrase IV
CAIX	carbonic anhydrase IX (protein)
*CA9*	carbonic anhydrase 9 (gene)
CAXII	carbonic anhydrase XII
cAMP	cyclin adenosine monophosphate
CRC	colorectal carcinoma
CREB	cAMP response element-binding protein
DTT	dithiothreitol
EGR	early growth response proteins
ER	endoplasmatic reticulum
ERE	estrogen response elements
ERK	extracellular signal-regulated kinase
ETS	proto-oncogene 1, transcription factor
FCS	fetal calf serum
FITC-CA-i	fluorescein isothiocyanate conjugated sulfonamide inhibitor
HGF	hepatocyte growth factor
HIF-1	*hypoxia-inducible factor 1*
HRE	hypoxia response element
HRP	horseradishperoxidase
HUVEC	human vascular endothelial cells
IAA	iodoacetamide
IC	intracellular
IPTG	isopropyl β-d-1-thiogalactopyranoside
I-TASSER	Iterative Threading ASSEmbly Refinement
MAPK	mitogen-activated protein kinase
NBCe1	sodium bicarbonate cotransporter 1
NFκB	nuclear factor kappa B
PI3K	phosphatidylinositol-3-kinase
PIMT	protein-L-isoaspartyl methyltransferase (protein)
*Pcmt1*	protein-L-isoaspartyl methyltransferase (gene)
PKA	protein kinase A
PLA	proximity ligation assay
PLGS	ProteinLynx Global Server
RET	tyrosine-protein kinase RET
S100A4	S100 calcium binding protein A4
SP1	specificity protein 1
SRC	tyrosine-protein kinase SRC
TF	transcription factor
TM	transmembrane
wt	wild type
XRE	xenobiotic response elements
